# Nitrogen Assimilation, Abiotic Stress and Glucose 6-Phosphate Dehydrogenase: The Full Circle of Reductants

**DOI:** 10.3390/plants5020024

**Published:** 2016-05-11

**Authors:** Sergio Esposito

**Affiliations:** Dipartimento di Biologia-Università di Napoli “Federico II”, Complesso Universitario di Monte Sant’Angelo, Via Cinthia 4, Naples 80126, Italy; sergio.esposito@unina.it; Tel.: +39-081-679124; Fax: +39-081-679233

**Keywords:** oxidative pentose phosphate pathway, redox regulation, nitrogen assimilation, abiotic stress

## Abstract

Glucose 6 phosphate dehydrogenase (G6PDH; EC 1.1.1.49) is well-known as the main regulatory enzyme of the oxidative pentose phosphate pathway (OPPP) in living organisms. Namely, in Planta, different G6PDH isoforms may occur, generally localized in cytosol and plastids/chloroplasts. These enzymes are differently regulated by distinct mechanisms, still far from being defined in detail. In the last decades, a pivotal function for plant G6PDHs during the assimilation of nitrogen, providing reductants for enzymes involved in nitrate reduction and ammonium assimilation, has been described. More recently, several studies have suggested a main role of G6PDH to counteract different stress conditions, among these salinity and drought, with the involvement of an ABA depending signal. In the last few years, this recognized vision has been greatly widened, due to studies clearly showing the non-conventional subcellular localization of the different G6PDHs, and the peculiar regulation of the different isoforms. The whole body of these considerations suggests a central question: how do the plant cells distribute the reductants coming from G6PDH and balance their equilibrium? This review explores the present knowledge about these mechanisms, in order to propose a scheme of distribution of reductants produced by G6PDH during nitrogen assimilation and stress.

## 1. The Roles of OPPP and Its Regulation by G6PDH in Planta

The oxidative pentose phosphate pathway (OPPP) is an almost ubiquitous pathway present in all Eukarya and most Bacteria [1]; only Archea appear to be missing a complete OPPP [2].

**Figure 1:**
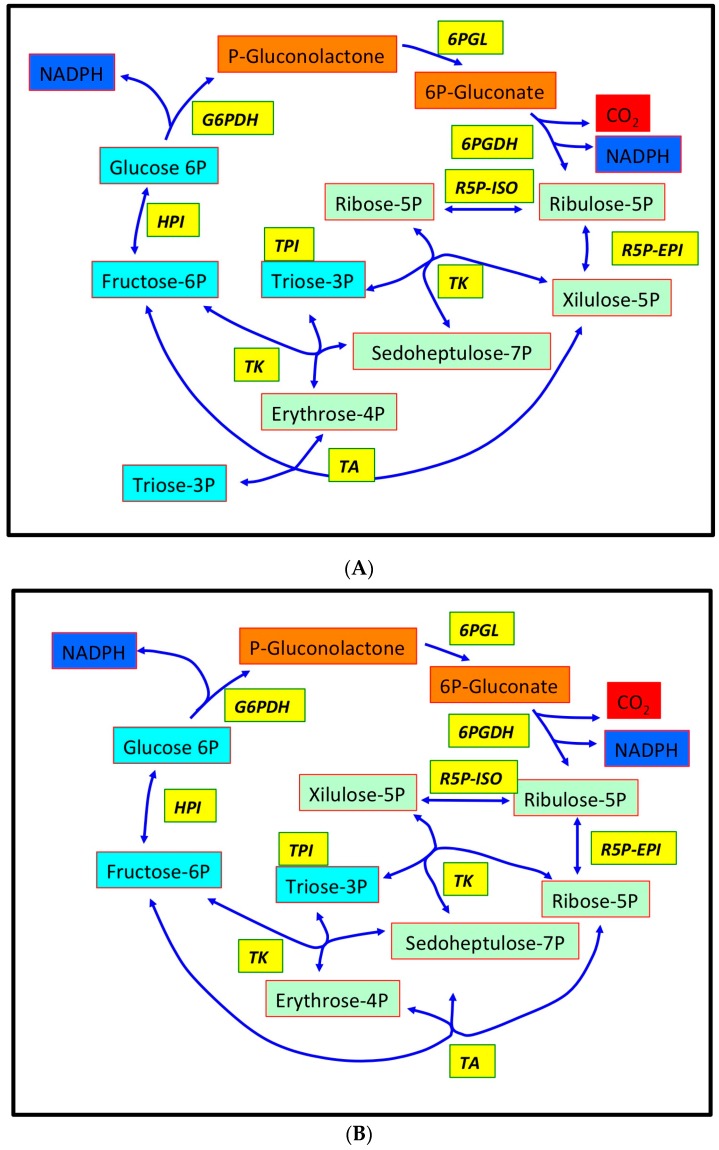
Design of conventional (**A**) and alternative (**B**) oxidative pentose phosphate pathway (OPPP) according to [1]. Intermediates peculiar of the OPPP are highlighted in orange; intermediates common to glycolysis are highlighted in light blue; intermediates common to Calvin–Benson cycle are highlighted in light green. Enzymes are highlighted in yellow. CO_2_ evolved is highlighted in red and NADPH reduced in blue. To avoid confusion, the inter-conversion of glyceraldehyde-3P and di-hydroxy-acetone-P by triose phosphate isomerase (TPI; EC 5.3.1.1) is omitted. List of enzymes abbreviations: G6PDH, Glucose-6-phosphate 1-dehydrogenase (EC 1.1.1.49); 6PGL, 6-Phosphogluconolactonase (EC 3.1.1.31); 6PGDH, 6-Phosphogluconate dehydrogenase (decarboxylating) (EC 1.1.1.44); R5P-ISO, Ribose-5-phosphate isomerase (EC 5.3.1.6); R5P-EPI, Ribulose-5-phosphate 3-epimerase (EC 5.1.3.1); TA, Transaldolase (EC 2.2.1.2); TK, Transketolase (EC 2.2.1.1); HPI, Hexose-6-phosphate isomerase (EC 5.3.1.9). Modified from [1].

The whole process consists of the oxidation of glucose-6-phosphate (G6P) to pentose-P, by evolving one CO_2_ molecule and reducing two molecules of NADP^+^ to NADPH (Figure 1); it has been estimated that 15% to 30% of hexose phosphate oxidized to glyceraldehyde-3P in a plant cell is processed by OPPP.

The whole pathway has been considered for a long time to play pivotal role in cell metabolism, representing the central point of many cellular processes, such as the supply of carbon skeletons for nucleotide synthesis; and, as in all eukaryotic cells, being a major source of NADPH; OPPP is critical to maintain redox balance under stress situations, e.g., during cell proliferation, in ageing, and in cancer cells [3].

Part of the intermediates of OPPP (glyceraldehyde-3P and fructose-6P) are shared with glycolysis, and, in photosynthetic organisms, some others (e.g., erythrose-4P and pentose-P) are common to the Calvin–Benson cycle as well (Figure 1); moreover, in plant cells, a not-secondary role is given by the furnishing of E4P and triose-P for shikimate pathway during chlorophyll biosynthesis.

This generates a very complex web of metabolic pathways, apparently not strictly related, and on the other hand, still far from being fully elucidated in their regulation, relative activities, and fluxes.

The first part of the OPPP can be described as a characteristic irreversible oxidative phase, in which CO_2_ has evolved from G6P, and NADP^+^ reduced to NADPH; a following reversible part re-generates hexose-P starting from pentose-P.

The initial reactions are carried out by glucose-6P dehydrogenase (EC 1.1.1.49-G6PDH), producing 6-phospho-glucono lactone, which is then hydrolyzed by a 6-phosphogluconolactonase (6PGL-EC 3.1.1.31) to 6-phosphogluconic acid; successively, a phosphogluconic acid dehydrogenase (EC 1.1.1.44-6PGDH) detaches a CO_2_ molecule, reducing a NADP^+^ to NADPH, thus forming ribose-5P. These reactions are regulated and limited by the first step: G6PDH reaction is the controlling enzyme of the whole pathway, given that its activity is able to pace the full cycle rate (Figure 1).

The following steps of the regenerating phase are played by ribulose-5P epimerase, ribose-5P isomerase, transketolase and transaldolase, reconverting different three- to seven- (or possibly eight-) carbon phosphorylated sugars; all of these reactions are generally considered near to equilibrium [1].

In plants, the OPPP subcellular localization suggests a complex network of coordination of carbon pathways in cells: although the cytosolic OPPP cycle represents the major part of the measured activity (about 60%–85% of the total, [4]), the existence of a complete OPPP confined in the plastidial compartment has been widely demonstrated.

Interestingly, this scheme is not always confirmed in other organisms possessing photosynthesis, such as Diatoms, where only the cytosolic OPPP is present, and most of the enzymes of the pathway are absent by these peculiar plastids [5].

It should be emphasized that many studies have demonstrated that, in plant cells, the cytosolic OPPP is not always complete, and, apart from G6PDH and 6PGDH, some of the enzymes of the regenerative segment of the pathway might be missing in the cytosol [6]. On the other hand, relative to compartmented OPPP, at least two distinct G6PDHs are present in higher plants, with different regulatory properties, thus suggesting that chloroplasts, and heterotrophic plastids could be equipped with differently modulated OPPPs.

## 2. G6PDH Isoforms in Plants

Plants’ G6PDH is an active homotetramer (200–250 kDa) or homodimer (100–120 kDa) formed by subunits (50–60 kDa). The assembly of the subunits is possibly played by saline bridges as described for human G6PDH: this structure is stabilized by NADP^+^; when the NADP^+^/NADPH ratio is low, the enzyme splits into its inactive monomers [7].

**Figure 2:**
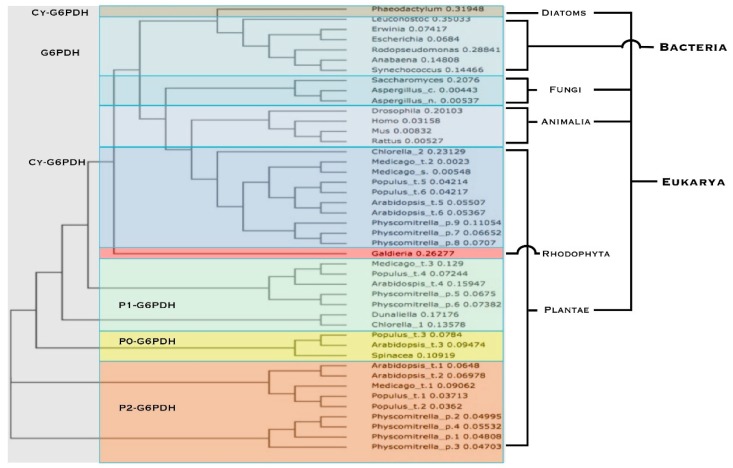
Phylogenetic tree of G6PDH isoforms from various living organisms. Blue and violet highlights designate cytosolic isoforms (simply indicated as G6PDH in Bacteria). Green highlight indicates the chloroplastic P1 isoforms. Yellow indicates the catalytically inactive P0-isoforms. Orange for the plastidial P2-G6PDH isoforms. Red highlight is for Red Algae isoform. Other details in the text.

The comparison of 44 amino acid sequences encoding for G6PDH from Bacteria, algae, Fungi, Metazoa and Planta, generates a phylogenetic tree organized in different main branches: the first comprises G6PDHs from Procarya, diatoms, Fungi and animals, localized in the cytosol; an adjacent branch with all the cytosolic isoforms from plants. A second main branch, clustering all the plant plastidic G6PDHs, splits in four different arms: P1-G6PDH (chloroplastic isoform), green algae chloroplastic isoforms, P0 isoforms (its role will be discussed later) and finally P2-G6PDHs (plastidic isoform) (Figure 2).

It is interesting to underline the distant position of Diatoms’ G6PDH and Rhodophyta’s G6PDH in this tree: it is worth remembering that Heterokontophyta chloroplasts originated by a secondary endosymbiosis event with a red alga (or a strictly related unknown organism). In Diatoms, the OPPP is solely cytosolic [5]; the *Galdiera* G6PDH sequence here reported is located in the chloroplasts [8], but clustered with cy-G6PDHs (even if very close to Plantae P1-G6PDH) (Figure 2).

Commonly, the G6PDH sequence presents the typical motifs of dehydrogenases: A Rossman-fold motif on the N-terminus, and NADP^+^-binding site were found. The putative active region (YRIDHYLGKE) presents a tyrosyl-residue, which is distinctive of cytosolic isoforms, instead of a phenylalanine, characteristic of compartmented isoforms.

### 2.1. Cytosolic G6PDH (Cy-G6PDH)

Generally, in higher plants at least two cytosolic isoforms are present, differently expressed in various tissues [9,10].

The biochemical properties of cy-G6PDH have been studied in potato [11] and in barley (*Hordeum vulgare*), both on purified enzyme from roots [12] and recombinant enzyme [13].

The cytosolic G6PDH generally shows a low sensitivity to reducing power; the enzyme is regulated by NADPH/NADP^+^ ratio and it is competitively inhibited by NADPH [11,13].

A regulation of cytosolic isoform activity by sugar-sensing mechanism has been suggested: this would control root nitrogen and sulfur assimilation on dependence of carbon status of the plant, in order to manage the amino acid synthesis [13].

Recent studies demonstrate the activation of cy-G6PDH from *Arabidopsis. thaliana* (*A. thaliana*) is controlled by the phosphorylation of Thr-467 by Glycogen Synthase Kinase 3 (ASKα) upon salt stress to sustain the increased request of reductants [14].

### 2.2. Chloroplastic G6PDH (P1-G6PDH)

The presence of a G6PDH activity confined in chloroplasts has been widely demonstrated [15,16]: this chloroplastic enzyme, generally defined as P1-G6PDH, is reversibly inhibited by light to guarantee an efficient photosynthesis, and avoid a futile cycle; in the dark this inhibition is removed, and the OPPP is activated to produce reducing equivalents [16].

Light inhibition of the chloroplastic G6PDH is explained by the redox modifications through the ferredoxin/thioredoxin system [1]: it has been demonstrated that this regulation is carried out by at least two cysteine residues on the N-terminus of the active protein sequence [17].

Chloroplastic P1-G6PDH has been shown to be highly sensitive to NADPH (Ki_NADPH_ < 8 μM), acting as a competitive inhibitor [11,18]; moreover, the chloroplastic isoform is controlled by a number of other factors, such as NADPH/NADP^+^ ratio, Mg^++^ and ribulose-5P [4,16].

### 2.3. Plastidial G6PDH (P2-G6PDH)

A second G6PDH isoform is detectable in plant roots upon nitrogen supply; this activity is, at least in part, responsible of the doubling of OPPP rate in root tissues; molecular studies confirmed the existence of this second, plastidial enzyme [12,19,20].

This plastidial G6PDH (referred as P2-G6PDH) is expressed in nearly all plant organs [9,21], and it exhibits distinct kinetic properties with respect to both cytosolic and chloroplastic G6PDHs [12,20,21,22]. The activity of this isoform is tightly modified upon reduction possibly in a similar way as for P1-G6PDH, even if the possibly of a specific thioredoxin class interacting with P2-G6PDH could be suggested [23,24].

A detailed kinetics study indicated that plastidial P2-G6PDH reaction follows an ordered sequential mechanism [25], and that this activity is primarily regulated by the NADPH/NADP^+^ ratio, [12,26]; interestingly, the different sensitivity to NADPH represents the peculiar difference between chloroplastic and plastidial G6PDHs: P2-G6PDH is considerable refractory to inhibition by NADPH, showing a Ki_NADPH_ > 40 μM, a value 5–10 fold higher with respect to the values known for cytosolic and chloroplastic isoforms [4].

### 2.4. The Enigmatic P0-G6PDH

A genome wide analysis in *A. thaliana* depicted a complex pattern for G6PDH isoforms in higher plants, with genes encoding for one chloroplastic, two plastidic, two cytosolic G6PDH; to those fully functional encoding sequences, a singular gene (named G6PD4) encoding for a non-functional enzyme was identified [9]. At the time, this was classified as a pseudo gene, but further research found an important role for this protein, now described as P0-G6PDH.

On the other hand, previous findings intriguingly suggested a possible presence of the OPPP in the peroxisomes [27,28]. Recently, it has been elegantly demonstrated that, upon oxidative stress, there is an assembly in the cytosol of P1-G6PDH subunits with P0-G6PDH [29]: These hetero-dimers are able to enter the peroxisomes, due to the presence, in the C-terminal of P0-G6PDH, of a peroxisome targeting sequence (PTS), exposed for preferential targeting in these organelles. Similarly, specific 6PGL and 6PGDH have been recently localized in *Arabidopsis* peroxisomes: this would create an efficient NADPH producing machinery in the peroxisomes upon stress or for possible specific NADPH requirement in these organelles [28,29,30,31].

### 2.5. The Subcellular G6PDH Localization Is Not Obvious in Plant Cells

Despite of the categorization of the different isoforms, many evidences would suggest that the subcellular and tissue localization of G6PDHs in plant cells are not constant, or obvious. As a major example, in *A. thaliana*, the chloroplastic (P1-G6PDH, encoded by G6PD1), and one of the two plastidial P2-G6PDHs (encoded by G6PD2) show kinetic properties that are opposite of those described for all the other plants studied so far, G6PDH1 exhibiting a low NADPH sensitivity, and G6PDH2 a high sensitivity to NADPH [9].

Further, the exclusive occurrence of P1-G6PDH in chloroplasts, which has been described without uncertainness for decades, has been disputed in the last few years for the newly demonstrated interaction with P0-G6PDH [29,31].

Recent studies still debate about the localization of plastidial isoform: transcript analysis demonstrated that P2-G6PDH is expressed in roots and non photosynthetic tissues (as expected) in potato and *Arabidopsis* [21]. On the other hand, both transcripts and abundance of P2-G6PDH could be detected, even if a lower extent, in green tissues [4,21]. At moment, it is far from being demonstrated if this isoform present in the leaves really occurs in photosynthetic cells or in different tissues of the leaves (e.g., epidermis, stomata, phloem, *etc.*).

## 3. G6PDHs and Nitrogen Assimilation

The main route of nitrogen assimilation in plant tissues involves the glutamine synthetase (GS; EC 6.1.1.3)/glutamate synthase (ferredoxin [Fd]-GOGAT; EC 1.4.7.1; NADH-GOGAT; EC 1.4.1.14) cycle [32].

In higher plants, GS is present as a plastidial enzyme, and several cytosolic isoforms are differently distributed in tissues. On the other hand, Fd-GOGAT and NADH-GOGAT isoforms are both located in the plastids. In leaf tissues there is the bulk of Fd-GOGAT activity, involved in photorespiratory ammonia assimilation; NADH-GOGAT is the major isoform in the roots, and is involved in the primary nitrogen assimilation [32].

The relationships between carbon metabolism and nitrogen assimilation have been widely studied, and many studies concerned the effects of ammonium on respiration and OPPP [4,19,20,26,33].

The reduction of nitrate to ammonium and the formation of glutamate both require reductants, supplied by photosynthetic process in the light; in the dark, and in heterotrophic tissues, the oxidative processes generate the reducing power and ATP.

Nitrite reductase requires ferredoxin as electron donor; in barley roots ferredoxin-ADP^+^ reductase differs from its leaf counterpart [33]: As consequence, high levels of NADPH are not essential to reduce ferredoxin, suggesting that G6PDH plays a central role during nitrate assimilation in heterotrophic tissues [19]. Intriguingly, both G6PDH and 6PGDH activities increased under nitrate assimilation, strongly suggesting a coordination between the nitrogen metabolism and OPPP [34].

Conclusively, nitrite reduction is directly connected to CO_2_ evolution from C1 of G6P, clearly indicating that OPPP is activated during nitrogen assimilation [19].

Overwhelmingly, the promoter sequences of nitrite reductase, FNR, ferredoxin and G6PDH all present the same NIT-2 motif, a nitrogen metabolism regulating factor [35], confirming the molecular coordination of the expression of both G6PDH and enzymes and proteins directly involved in nitrogen metabolism.

The tight relationship between OPPP and nitrogen metabolism is not confined within nitrate reduction process: GOGAT activity is clearly supported by plastidic G6PDH, which is able to satisfy the increased request of reducing power upon ammonium assimilation.

In N-starved barley roots, only the cytosolic isoform can be detected by Western blotting, [4,26], and a reduced expression of the plastidic isoform was observed [34]; supply of nitrogen results in a prompt increase of G6PDH activity, partially ascribing to the a newly synthetized plastidial isoform [4,26].

Further evidence of the strict correlation between plastidial OPPP and GOGAT activity has been given by experiments on isolated organelles: root plastids are able to synthesize glutamate using GOGAT only if substrates of the OPPP (e.g., G6P or pentose-P) are present [19,20]; the kinetic of the G6P-dependent glutamate synthesis in root plastids suggested that G6PDH is able to support maximal levels of glutamate synthesis even under sub-saturating conditions [19,20].

Based on the evidences given by a number of papers, a general scheme for the strict relationship between G6PDHs and GOGAT isoforms can be hypothesized: nitrogen caused an increase of NADH-GOGAT activity in the roots [4]; the reducing power in these tissues for NADH-GOGAT is likely furnished by P2-G6PDH, which is in turn induced by nitrogen (possibly under the stimuli of glutamine levels, [26]).

The low sensitivity to NADPH inhibition would help to sustain P2-G6PDH for the supply of reductants during nitrogen assimilation in the roots; and in the light/dark transition in the leaves [4,34,35,36].

In the leaves, P1- and P2-G6PDHs would supply reducing power for Fd-GOGAT enzyme, in the first dark phase, when the halt in photosynthesis produces high ammonium levels due both to photorespiration and deamination of glutamine and asparagine [34,37].

Intriguingly, it could be argued that-upon high nutrient availability-the ability of P2-G6PDH to maintain part of its activity under high NADPH/NADP^+^ ratios would allow the support the nitrogen assimilation in the light [3,4,36].

The strict relationship between OPPP and nitrogen metabolism has been recently investigated by a genetic approach, demonstrating that N assimilation genes are expressed by a sugar sensing mechanism, in its turn requiring OPPP activity. Therefore, the expression of nitrate assimilation genes in the nucleus of root cells is promoted by a signal emanating from OPPP activity in the plastid [38].

## 4. G6PDHs upon Abiotic Stress

It has been suggested that the early response to the abiotic stress would involve the OPPP [14,39,40,41], which represent a true metabolic sensor during response to oxidative stress [42]. The possible physiological and biochemical roles played by OPPP, and namely G6PDH, in plant tissues during different stress have been investigated [29,43,44]: Changes in OPPP have been demonstrated upon cold stress [45,46], heat [47], metals pollution [48,49,50,51], drought [52,53] and salinity [18,54,55].

Furthermore, many evidences suggest that this role in the response to oxidative stress can be widened to pathogens attack [56,57,58,59], but this will not be discussed in this review further.

First evidences for a direct G6PDH involvement in stress response were given examining short-term genes involved in the response to salinity in wheat [54]; among these WESR (Wheat Early Salt Responding) genes, WESR5 had been identified as encoding for a cytosolic G6PDH [41].

The increase of both expression and activity of G6PDH has been extensively described during salt stress [18,43,44,58] which presumably activate the whole set of the enzymes of OPPP [54]. Salinity caused an increase of G6PDH activity in barley roots, regardless of nitrogen supply; this increase is dependent on the de-novo synthesis of G6PDH, possibly ascribed to the cytosolic isoform; thus, it is evident that the increase of G6PDH activity induced by salt stress falls outside the normal pattern of physiological conditions [18].

This effect can be ascribed to an ABA signaling pathway, activating the ABA signaling cascade, and inducing those genes which present ABRE elements in the promoter region [60], such as G6PDH isoforms in wheat [54], tomato [53], rice [60], barley [13,39] and other plants. Correspondingly, in barley roots exposed to exogenous ABA root, P2-G6PDH occurrence and activity was enhanced [39], indicating an ABA-responsive pathway for G6PDH expression; this hypothesis has been recently confirmed in tomato, where analogous increases in expression of 9-cis-epoxycarotenoid dioxygenase (NCED — the ABA synthetizing enzyme), protein phosphatase 2C-type (PP2C—the target of the ABA receptors PYR-PYL/RCAR) are associated to cy-G6PDH expression, occurrence and activity, in both hydroponic, greenhouse and field growing conditions [53].

Furthermore, cy-G6PDH would usually able to maintain an adequate NADPH/NADP^+^ ratio in the cytosol but, under severe stress conditions, a diversion of NADPH produced for nitrogen metabolism by P2-G6PDH does occur, in order to counteract the dangerous effects of stress, thus halting nutrient assimilation [18]. Transgenic tobacco overexpressing a plastidic G6PDH exhibited no visible phenotype, but showed metabolism alterations, suggesting that P2-G6PDH may be essential in balancing the redox charge in organelles [39].

Different modifications of G6PDH isoforms, affecting both activity and kinetic properties upon stress could have been proposed: chaperonins action, sugar signaling and phosphorylation [14,61,62].

OPPP has been suggested as a major pathway involved in the sugar induction of nitrogen (and sulfur) carriers in plant roots. This signaling pathway would coordinate nutrient uptake, and their assimilation, together with the providing of NADPH necessary for amino acid synthesis [61].

Salt stress activates the synthesis of Glycogen synthase kinase 3 from *A. thaliana* (ASKα); in its turn, ASKα is able to phosphorylate cy-G6PDH on Thr-467, thus stimulating the catalytic activity, and enhancing NADPH production to counteract ROS excess [14]; therefore the antioxidant system is able to reduce H_2_O_2_ to water by the glutathione peroxidase cycle or by the ascorbate-glutathione cycle [53].

## 5. How Does the Plant Cell Cope with the Distribution of Reductants Produced by G6PDH?

All these recently discovered patterns of regulation—and subcellular localization—of different G6PDH isoforms shed a new light in the emerging complex role of this pivotal enzyme in the furnishing of reductants for both nutrient assimilation and stress response in plant cells.

Thus, a general picture of the involvement of OPPP in the response to abiotic stress in plant cells can be designed: the whole pathway would be able to provide reductants to counteract the effects of stress (e.g., ROS production); of course, due to its central role in nutrient assimilation, this action would be exerted by diverging the reductants physiologically utilized during nitrogen assimilation. Therefore, abiotic stress conditions would cause a partial but consistent distraction of NADPH- produced by the OPPP- from basal metabolism to ROS scavenging [22,46].

Anyway, how can the reductants produced by G6PDH be addressed towards nitrogen metabolism, or stress response? How could this mechanism be triggered?

The oxidative damage induced by stress increased NADPH synthesis by OPPP; it can be hypothesized that the need to maintain of steady-state level of H_2_O_2_ in cells during stress would allow the function of hydrogen peroxide as a signal inducing an enhanced G6PDH expression and activity [63].

Furthermore, the G6PDH involvement in stress response could intersect nitrogen metabolism in a more complex pattern. Nitric oxide (NO) has been demonstrated as involved in responses to most of stress in plant cells [64,65]; among the different pathway synthetizing NO in plants, a preferential role is played by nitrate reductase (NR), whose activity is directly enhanced by G6PDH upon salinity in roots [66]. It has been proposed that G6PDH would increase NADPH levels stimulating the NR-dependent NO production, thus enhancing the activities of antioxidant pathways, in order to scavenge the ROS induced by salt stress [64,66]; a similar mechanism has been suggested in soybean roots during cadmium stress [49].

Of course, a question raises about how the plant cell deals with the diversion of reductants and the increase of G6PDH activity: which are the fast-responding factors connecting OPPP to abiotic stress and, at the same time, able to turn off (or at least lowering) the supply of reductants for nitrogen metabolism?

Recent studies strongly suggest that thioredoxins could play the role of regulators of G6PDH activity. Even if this has been known for decades, and widely recognized, recent studies indicate that the different G6PDH isoforms are not all regulated by the same thioredoxins, and often their activity can me modulated by single specific class (or sub classes) of TRXs at a different extent [23,24,67,68]. Namely, P1-G6PDH appears to be modulated by TRX *f* [23,67]; it has been previously suggested Trx *m* as a modulator of P2-G6PDH activity, but this was obtained using crude bacterial extracts overexpressing his tagged recombinant protein [17]; recently, a specific modulation by TRX *m*, and not TRX *f*, of highly purified recombinant P2-G6PDH from *Populus trichocarpa* has been suggested [69].

Thus, TRXs could represent a highly flexible system to modulate the activity of the different G6PDH isoforms, (and possibly their subcellular localization as well), resulting in the optimal distribution of reductants produced by the plant cell.

**Figure 3:**
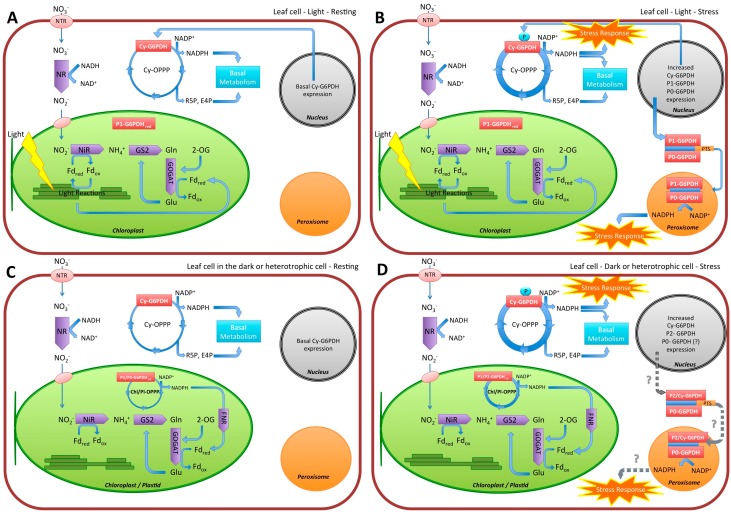
The fate of reductants derived from Glucose-6P dehydrogenase (G6PDH), and more generally Oxidative pentose phosphate pathway (OPPP), in plant cells. (**A**). In photosynthetic cells, cytosolic OPPP provides NADPH for basal metabolism in the light. Normally, photosynthesis is able to sustain the request of electrons for nitrogen metabolism. Chloroplastic P1-G6PDH results inhibited by NADPH and thus OPPP is inactive in the organelles. (**B**). In the leaves, under stress conditions, there is an increase of cytosolic OPPP in order to supply NADPH for stress response. An increased expression of cy-G6PDH occurs, together with expression and synthesis of P1- and P0-G6PDH: this causes the formation of heterodimers directed to peroxisomes; there is the activation of a specific machinery formed by oxidative section of OPPP to counteract the stress; or for different metabolic functions in specific plant organs. (**C**). In the dark, or in heterotrophic tissues—under physiological conditions—chloroplastic (or plastidial) OPPP are activated by P1-G6PDH or P2-G6PDH, respectively, providing reductants for nitrogen assimilation in the dark/heterotrophic conditions. As in leaves, cytosolic OPPP provides NADPH for basal metabolism. (**D**). In the dark, or in heterotrophic tissues, under stress there is an increase of both cytosolic OPPP (by cy-G6PDH), and chloroplastic/plastidial OPPPs (by P1-G6PDH or P2-G6PDH, respectively), in order to counteract the stress. It is possible to hypothesize (?) the formation of heterodimers directed to peroxisomes (dotted grey arrows), but this has not been proven yet. It must be underlined that P2-G6PDH is able to maintain a high rate even at NADPH/NADP^+^ ratios normally easily inhibiting P1-G6PDH, thus sustaining the stress response in root/heterotrophic tissues (e.g. enduring drought/salt stress conditions). Abbreviations: NTR, nitrate transporters; NR, Nitrate reductase; NiR, Nitrite reductase; GS, glutamine synthetase; GOGAT, glutamate synthase; Fd, Ferredoxin (red,reduced/ox, oxidated); R5P, ribose-5P; E4P, erythrose-4P; Glu, glutamate; Gln glutamine; 2-OG, 2 oxoglutarate; PTS, peroxisome targeting sequence.

A general scheme could be defined, taking in account the different regulatory mechanisms existing on this enzyme (Figure 3).

Under “normal” conditions (e.g., light, irrigation, nutrient availability optimal) NADPH produced by G6PDH can be utilized for nitrogen assimilation, e.g., by GOGAT and NiR (Figure 3A). When stress conditions appear, different signals trigger the diversion of NADPH from nitrogen assimilation to abiotic stress response (Figure 3B). Firstly, an ABA signaling pathway should be able do divert the reductants for ROS detoxification: this may occur both by moving heterodimers P1-P0 into peroxisomes, and increasing the activity of plastidial P2-G6PDH.

Further, a major mechanism would be represented by cy-G6PDH phosphorylation by Ask-α, specifically connected to plant stress response.

In the dark, or in heterotrophic tissues, NADPH is usually provided by OPPP in the cytosol (Figure 3C), and an enhanced G6PDH activity would support reductants increased request upon stress (Figure 3D). In chloroplasts in dark, and in non-photosynthetic plastids, P1-G6PDH, and P2-G6PDH would be able to counteract the effects of stress, respectively. At the moment, it could only be speculated that, similar to P1/P0-G6PDH hybrid enzymes, a re-direction of G6PDH could occur in peroxisomes of heterotrophic tissues, but this should be properly investigated in the future (Figure 3D).

## 6. Conclusions

In plant cells, G6PDH plays a central role in both providing reductants and precursors for basal metabolism, and to counteract the oxidative burst upon stress conditions. Therefore, G6PDH can be considered as a main factor of cell redox poise, determining sensitivity to stress.

The compartmented chloroplastic (P1-G6PDH) and plastidial (P2-G6PDH) isoforms are connected with the furnishing of reductants for nitrogen assimilation, but upon stress conditions, both activities can be recruited for resistance and/or tolerance strategies.

The interconnections between OPPP, nitrogen metabolism, and stress response are multiple: the ABA signaling pathway plays a central role in the induction of G6PDH upon stress and the reductants produced by OPPP can be diverted from nitrogen assimilation towards the ROS scavenging; moreover thioredoxins modulating activity of the different G6PDH isoforms could selectively trigger the different isoforms.

Furthermore, it should be remembered that, in higher plants, nitric oxide can be synthetized by nitrite reductase upon high levels of NADPH (increased upon stress) creating a loop between OPPP, nitrogen assimilation and NO signaling.

Last but not the least, the synthesis of P0-G6PDH upon stress would be able to form hybrid G6PDHs redirected in different organelles (e.g., peroxisomes) to counteract oxidative stress.

The definition of a crystal structure of plant G6PDH isoforms, and a more clear definition of the kinetic and inhibitory mechanisms are still required in order to correctly define their role in plant cell metabolism. Although in the last decade a huge leap has been done in the understanding of controlling mechanisms overlying G6PDH activity in plants, there is still a long path to reach the goal of a comprehensive definition of the regulation of this pivotal enzyme in plants.
